# Isolation, Characterization and IgE Binding of Two 2S Albumins of Pomegranate Seeds

**DOI:** 10.3390/foods13131965

**Published:** 2024-06-21

**Authors:** Lisa Tuppo, Claudia Alessandri, Laura Zaccaro, Ivana Giangrieco, Maurizio Tamburrini, Adriano Mari, Maria Antonietta Ciardiello

**Affiliations:** 1 Institute of Biosciences and BioResources (IBBR), National Research Council of Italy (CNR), 80131 Naples, Italy; lisa.tuppo@ibbr.cnr.it (L.T.); ivana.giangrieco@ibbr.cnr.it (I.G.); maurizio.tamburrini@ibbr.cnr.it (M.T.); 2 Associated Centers for Molecular Allergology (CAAM), 00100 Rome, Italy; claudia.alessandri@caam-allergy.com (C.A.); adriano.mari@caam-allergy.com (A.M.); 3 Institute of Biostructures and Bioimaging (IBB), National Research Council of Italy (CNR), 80131 Naples, Italy; laura.zaccaro@cnr.it

**Keywords:** protein biochemistry, food allergy, sensitization, seed storage proteins, protein purification, N-terminal amino acid sequencing, mass spectrometry, bioinformatics, immunoblotting, Italian population

## Abstract

Literature reports suggest that the presence of proteins in pomegranate seeds is responsible for sensitization and IgE-mediated allergic reactions. The objective of this study was the analysis of a pomegranate seed extract and the isolation and characterization of proteins contained in high amounts. The extract characterization showed a protein profile with main bands at about 18 kDa and below 10 kDa upon SDS-PAGE, and molecules were recognized by specific IgEs upon immunoblotting. Then, two new 2S albumins, a monomeric and a heterodimeric one, were isolated by using classical biochemical methods. They were identified via direct protein sequencing and mass spectrometry, and their primary structure was analyzed and compared with homologous allergenic proteins via bioinformatics. In an Italian population of 703 suspected allergic patients, analyzed by using the FABER^®^ test, the frequency of sensitization to the monomeric and heterodimeric 2S albumins was 1.7% and 0.28%, respectively. This study reports for the first time the isolation and characterization of two 2S albumins from pomegranate seeds. The clinical relevance of these molecules needs further investigation, for instance in populations having different exposures and allergy profiles.

## 1. Introduction

Pomegranate, *Punica granatum* L., is a species belonging to the family *Punicaceae*, subclass *Rosidae*. This fruit tree lives in temperate climates and is believed to be native to the region between Iran and northern India. Currently, it is mainly cultivated around the Mediterranean area, in Southern Asia, and in some countries of North and South America [1]. It is one of the oldest cultivated species among fruit trees. The edible parts of pomegranate are the arils, which are seeds covered by a red pulp, which is a juice sac. The arils are surrounded by the white mesocarp, which separates the arils from the fruit peel.

The consumption of this fruit and its juice is gaining worldwide popularity not just for its unique color and taste but also because it is reported to be rich in bioactive compounds with beneficial effects in relation to several diseases, such as inflammatory pathologies, cancer, diabetes, vascular diseases, osteoporosis, and bacterial and viral infections [2,3,4,5,6,7,8,9]. In the recent years, pomegranate seeds have been recommended as a food supplement or in cosmetic products [10,11,12,13,14]. However, sometimes its consumption has to be avoided because this fruit is also a source of molecules which can trigger allergic reactions.

The first allergic reaction to pomegranate was reported in 1991 by Igea et al. [15] and was proven by a double-blind oral challenge test on an 85-year-old woman, who displayed tongue angioedema after the fruit’s intake. Next, a bronchospasm after pomegranate ingestion was described in 1992 [16], and later, in 1999, a 29-kDa allergen (Table 1) was detected via IgE-immunoblotting with the sera of patients reporting angioedema, urticaria, abdominal pain and anaphylactic shock after pomegranate ingestion [17].

A 9 kDa LTP [9k-LTP] was identified in 2007 and registered by the World Health Organization and International Union of Immunological Societies (WHO/IUIS) as Pun g 1 [18]. Afterwards, four 9k-LTP isoforms were detected via 2D immunoblotting. These proteins showed different immunological properties and partial cross-reactivity with Pru p 3 [21]. The crystal structure of pomegranate Pun g 1 was also obtained and compared to homologous allergenic proteins, such as the gold kiwifruit LTP, Act c 10 [22].

In 2017, a 7 kDa allergen, named pommaclein, was isolated from the pomegranate red pulp, characterized and registered by WHO/IUIS as Pun g 7 [19]. It is homologous to the peach allergen, peamaclein (Pru p 7), a member of the gibberellin-regulated protein (GRP) family [23]. More recently, a 29 kDa protein belonging to the class III chitinases was identified in the pomegranate pulp and registered as allergen Pun g 14 [20]. The structural characterization of this allergen revealed that its amino acid sequence corresponded to chitinase III, was able to bind calcium ions with high capacity and low affinity, and was found in the pomegranate seed by Yang et al. in 2011 [24]. This observation suggests that Pun g 14 could be present both in the pomegranate pulp and seed. Nevertheless, the literature reports showed that the frequency of sensitization to pomegranate seed proteins was higher than that observed for Pun g 14, thus suggesting that chitinase III cannot be the only IgE-binding protein contained in this tissue [20]. An allergy to pomegranate seeds was reported in 1992 by Gaig and collaborators [16], who described the case of a 7-year-old IgE-dependent asthmatic child who showed a clinical reaction just moments after ingesting several pomegranate seeds. Recently, an IgE-mediated pomegranate seed allergy in a girl with multiple tree nut allergies in the United States has been reported [25]. Therefore, as highlighted by some literature reports, pomegranate seeds can cause sensitization [20] and allergic reactions [16,25].

Nevertheless, beyond chitinase III, which was identified in the pulp as an allergen [20], and present also in the seed [24], no allergens have been isolated and identified yet directly in the pomegranate seed. However, as reported above, some literature data suggest the presence of such molecules in this tissue. In this context, the objective of this study was the search for IgE-binding proteins in a pomegranate seed extract, in order to identify them and perform initial characterizations at the structural and immunological level. The analysis of these potential allergenic proteins might be of great interest for the safety of allergic patients, and for the improvement of allergy diagnostics. The results obtained in experiments of IgE immunoblotting allowed the description of the available pomegranate seed as a possible cause of sensitization. In addition, two new 2S albumins were isolated by using classical biochemical methods and characterized, in comparison with known allergenic homologs, via Sodium Dodecyl Sulfate-PolyAcrylamide Gel Electrophoresis (SDS-PAGE), N-terminal amino acid sequencing, mass spectrometry, bioinformatics and specific IgE binding by using the patient-Friendly Allergen nano-Bead aRray (FABER)^®^ test.

## 2. Materials and Methods

### 2.1. Materials

Pomegranate fruits were purchased at the commercial ripening stage in a local market (Naples, Italy). Sodium Chloride was from VWR Chemicals (Leuven, Belgium). Phenylmethylsulfonyl Fluoride (PMSF) was from Boehringer (Mannheim, Germany). Ethylenediaminetetraacetic Acid (EDTA), Bovine Serum Albumin (BSA), Trifluoracetic Acid (TFA), ammonium bicarbonate and iodoacetamide were purchased from Sigma-Aldrich (Merck, KGaA, Darmstadt, Germany). Tris-(hydroxymethyl)-aminomethane (Tris) ultra-pure was purchased from ICN Biomedicals (Aurora, OH, USA). Diethylaminoethyl Cellulose (DE52) was from Whatman (Brentford, UK). Sulfopropyl-Sepharose (SP-Sepharose) was from Amersham Biosciences (Uppsala, Sweden). Bovine trypsin and Dithiothreitol (DTT) were from Roche Diagnostics (Mannheim, Germany). Acetonitrile and Methanol were from Carlo Erba Reagents srl (Milan, Italy). Acetic Acid was from Romil Ltd. (Deltek srl, Naples, Italy). A Bio-Rad Protein Assay, Coomassie R-250 brilliant blue, Polyvinylidene difluoride (PVDF) membranes and 5-bromo-chloro-3-indoyl phosphate p-toluidine salt/p-nitro blue tetrazolium chloride (BCIP/NBT) were from Bio-Rad (Milan, Italy). All other reagents were of the highest commercially available quality.

### 2.2. Methods

#### 2.2.1. Pomegranate Seed Extract Preparation

Pomegranate arils were manually separated into pulp and seeds. The seeds contained in a fruit (about 20 g) were used to prepare a protein extract. They were crushed with a pestle in a mortar and the powder was resuspended by adding 1 M NaCl (1:5 *w*/*v*), 0.05 mg/mL PMSF and 5 mM EDTA. After stirring for 2 h, the homogenate was centrifuged at 17,000× *g* for 45 min using a Sorval RC6 plus (Thermo Fisher Scientific, Osterode, Germany), and the supernatant, corresponding to the protein extract, was stored at −20 °C until use.

The protein concentration of the extract was determined by employing the Bradford method using the Bio-Rad Protein Assay, which is a colorimetric assay, by following the procedure suggested by the manufacturer. The experimental data, obtained as absorbance at 595 nm, were analyzed using as a reference a calibration curve made with BSA ranging from 2 to 10 μg.

#### 2.2.2. Purification of Two 2S Albumins (Pun g 2S-A1 and Pun g 2S-A2) from a Seed Extract

The extract was dialyzed against 10 mM Tris-HCl, pH 8.5, and then loaded on a hand-packed DE52 column (2.8 cm × 7.0 cm), equilibrated in the same buffer (buffer A). The acidic proteins bound to this resin (containing Pun g 2S-A1) were eluted via a linear gradient from 0% to 100% of buffer B (10 mM Tris-HCl pH 8.5, containing 0.5 M NaCl).

The protein fraction eluted in the column flow-through (containing Pun g 2S-A2), after lowering the pH to 7.2 via the addition of 6 M HCl, was loaded on a SP-Sepharose column (1.7 cm × 7.0 cm) equilibrated in 10 mM Tris-HCl, pH 7.2 (buffer A). The elution was carried out by using a linear gradient from 0% to 100% of buffer B (10 mM Tris-HCl, pH 7.2, containing 0.5 M NaCl) at a flow rate of 1 mL/min.

The obtained fractions were analyzed by using Reverse Phase-High Performance Liquid Chromatography (RP-HPLC) and/or SDS-PAGE, and then pooled. Further purification of both proteins was achieved via RP-HPLC on a Vydac C8 column (0.46 cm × 25 cm), using a Beckman (Fullerton, CA) System Gold apparatus. The elution was obtained via a multistep linear gradient of eluent B (0.08% TFA in acetonitrile) in eluent A (0.1% TFA in water) going from 1% to 60% in 60 min and from 60% to 90% in 10 min at a flow rate of 1 mL/min. The eluate was monitored at 220 nm and 280 nm. The peaks corresponding to each purified protein were manually collected, concentrated using a rotary evaporator Savant UVS450A-230 (Thermo Fisher Scientific, Asheville, NC, USA) and subjected to several washes with water to remove traces of TFA.

The purity of the protein preparations was assessed via SDS−PAGE, RP-HPLC and N-terminal amino acid sequencing. The protein concentration of the purified Pun g 2S-A1 and Pun g 2S-A2 was estimated on the basis of the molar extinction coefficient at 280 nm (9440 and 4970 M^−1^ cm^−1^, respectively) calculated by using the ProtParam tool, available on the Expasy Proteomics Server (www.expasy.org, accessed on several dates, including 17 May 2017 and 25 January 2024), using the amino acid sequence of the molecules having UniProt accession numbers A0A218XU94 and A0A2I0JHZ1, respectively, after removal of the signal peptide and ignoring the further proteolytic processing on the protein proform.

#### 2.2.3. Analysis via SDS−PAGE

The seed extract and purified 2S albumins were analyzed via 15% SDS−PAGE under reducing and non-reducing conditions on a Bio-Rad Mini Protean apparatus (Biorad, Segrate, Italy). The staining was carried out in 0.05% Coomassie R-250 brilliant blue in 40% methanol/10% acetic acid, and the rinsing was performed in 40% methanol/10% acetic acid.

#### 2.2.4. Amino Acid Sequencing

Both purified proteins were analyzed via N-terminal amino acid sequencing using the Edman degradation chemistry. In particular, 500 pmol was loaded on an automated Shimadzu protein sequencer, PPSQ-33B (Shimadzu Corporation, Tokyo, Japan). Moreover, native Pun g 2S-A1 was subjected to proteolytic cleavage by using bovine trypsin, following the manufacturer’s instructions. The digestion was carried out at an enzyme/protein ratio of 1:20 (*w*/*w*) in Tris-HCl 0.1 M pH 8.5 at 37 °C for 3 h. The obtained peptides were separated via RP-HPLC, manually collected, and some of them were analyzed via direct sequencing. The details about the RP-HPLC method are the same as already described in Section 2.2.2.

#### 2.2.5. Mass Spectrometry Experiments

The molecular mass of purified Pun g 2S-A1 was determined using a QSTAR^®^ Elite System equipped with a Nano-electroSpray^®^ Source (Applied Biosystems, San Diego, CA, USA). Spectral deconvolution and mass reconstruction were performed using the algorithm “Mass Reconstruction” in the Analyst QS 2.0 software (Applied Biosystems, Foster City, CA, USA). Its identification was performed using the method of enzymatic digestion and shotgun proteomics [26]. Briefly, 3.3 µg of native protein was solubilized in 0.1 M ammonium bicarbonate, containing 1 mM DTT and 5.5 mM iodoacetamide in 10% acetonitrile. Then, the sample was subjected to proteolytic digestion via bovine trypsin using an enzyme/protein ratio of 1:50 (*w*/*w*) at 37 °C for 20 h. The obtained peptides were separated on an UltiMate 3000 HPLC (Lc Packings) coupled directly with a QSTAR^®^ Elite System. MS/MS spectra were recorded by using the CID technique (collision-induced dissociation) and analyzed by using Analyst QS software. The identification was obtained using the MASCOT program on the website http://www.matrixscience.com (accessed on 17 May 2017) searching in the Plant Est_129 (164069424 sequences) database.

The mass spectrometric analysis of the protein, Pung g 2S-A2, previously purified and its subunits were carried out using the LC/MS LTQ XL™ Linear Ion Trap Mass Spectrometer (Thermo Scientific, Waltham, MA, USA) equipped with an electrospray ionization source and connected with an HPLC system. The sample was analyzed on the column Aeris WidePore (Phenomenex) 150 mm × 2.1 mm, 3.6 Å applying a gradient of eluent B (0.1% TFA in acetonitrile) in eluent A (0.1% TFA in water) from 5% to 80% in 30 min at a flow rate of 200 µL/min.

For the analysis of subunits, the native Pun g 2S-A2 was reduced by adding a stock solution of 1 M DTT until the final concentration was 14 mM. The solution was incubated for 30 min at 60 °C, and then the sample was analyzed via LC/MS using the conditions reported for the intact protein. The ESI spectra were deconvoluted by using the MagTran 1.03b2 software to yield the monoisotopic mass of the intact protein and its subunits.

#### 2.2.6. IgE Immunoblotting Analysis

The IgE binding capacity of the seed extract was detected via immunoblotting, using the sera described in a preceding paper [20]. The experiments were carried out following an already reported procedure [27]. Briefly, 25 µg of the extract was separated via 15% SDS−PAGE, transferred onto PVDF membranes and stained with ponceau red. After removal of the blocking solution, the membranes were incubated with sera of allergic subjects diluted 1:5, containing a specific IgE as the primary antibody. Next, a mouse monoclonal anti-human-IgE conjugated to alkaline phosphatase (diluted 1:1000) was used as a secondary antibody (Becton Dickinson Biosciences, San Jose, CA, USA). Immunoreacting allergen bands were revealed via incubation with BCIP/NBT solution prepared according to the manufacturer’s instructions.

#### 2.2.7. Specific IgE Detection Using the FABER^®^ Multiplex Testing System

The FABER^®^ test (ADL S.r.l., Latina, Italy) is a multiplex in vitro serological test allowing the detection of specific IgE antibodies from allergic subjects [28,29,30]. The version used to perform the present study (FABER^®^ 244-122-122) bears 244 allergenic preparations, including 122 purified molecular allergens and 122 allergenic extracts, all conjugated to nanobeads. The data were obtained from a set of biochips including pomegranate 2S albumins, spotted for experimental purposes. Before the immobilization on the FABER^®^ biochip, 2S albumins were coupled to nanobeads following the same procedure applied to all the standard allergens. As usual, the quality of each batch of FABER^®^ biochips, bearing the spotted allergens, was verified before use.

#### 2.2.8. Patients

Ten patients highly reactive to pomegranate were selected on the basis of a clinical history of allergy to this fruit or a reported refusal to eat it. The sera of these patients were used to perform an immunoblotting analysis shortly after collection, thus avoiding a long storage time. The characteristics of these sera are described in Tuppo et al. 2018 [20].

Later, 703 Italian unselected patients reporting any type of allergy were tested for the presence in their sera of IgE specific for purified pomegranate seed proteins using the FABER^®^ test (ADL S.r.l., Latina, Italy). The results were real-time transferred from the laboratory into the InterAll software (version 5.0, ADL S.r.l, Latina, Italy), which is a customized electronic record for diagnostic and clinical data storing.

#### 2.2.9. Bioinformatic Investigations

Primary structure similarity search in the UniProt, using the BLAST program, was performed on the server, www.expasy.org. The details about the protein molecules, including the molecular mass calculation on the basis of the primary structure, were obtained using the ProtParam tool (https://web.expasy.org/protparam/), on the Expasy platform (accessed on several dates from 17 May 2017 to 25 January 2024). The predicted cleavage sites obtainable via enzymatic digestion of purified Pun g 2S-A1 were determined through PeptideCutter on the expasy.org server. The allergen isoforms to be aligned for structural comparison were analyzed using the AllergomeAligner tool (www.allergome.org/script/tools.php?tool=blaster), on the Allergome platform (accessed on several dates from 17 May 2017 to 25 January 2024). The default parameters were used for all the bioinformatic analyses.

## 3. Results

**Figure 1:**
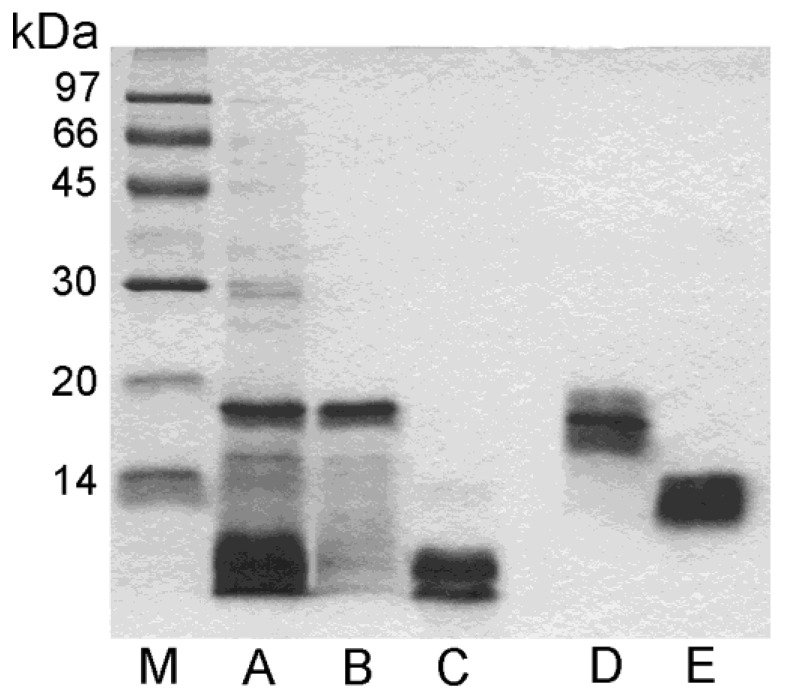
SDS-PAGE analysis under reducing (M, A, B, C) and not reducing (D, E) conditions. A, 25 µg of seed extract; B and D, 6 µg of purified Pun g 2S-A1; C and E, 6 µg of purified Pun g 2S-A2; M, molecular mass markers.

The pomegranate seed extract, prepared as reported in the Materials and Methods section, showed a total protein amount of 332 mg, which is about 16.6 mg/g of seeds. The protein profile obtained via reducing SDS-PAGE is shown in Figure 1.

### 3.1. Analysis via Immunoblotting of Pomegranate Seed Extract

**Figure 2:**
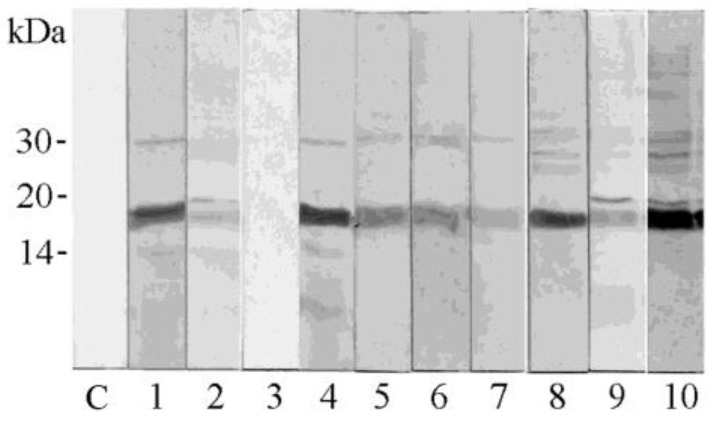
IgE immunoblotting experiments. The pomegranate seed extract was probed using ten individual sera (Lanes 1–10) and with buffer as negative control for anti-human IgE antibody (lane C).

Ten sera of patients reporting allergic reactions to pomegranate were used to probe the seed extract via IgE immunoblotting (Figure 2).

Nine sera showed a common band of a protein migrating at an apparent molecular mass of about 18 kDa. This band was very strong on the strips probed using sera 1, 4, 8 and 10. In addition, weak bands at different molecular masses could be noted in the immunoblotting. These results encouraged the isolation and identification of the main proteins contained in the extract to investigate the molecules possibly responsible for IgE binding and the allergy to pomegranate seeds.

### 3.2. Isolation of Two Proteins Contained in The Seed Extract

**Figure 3:**
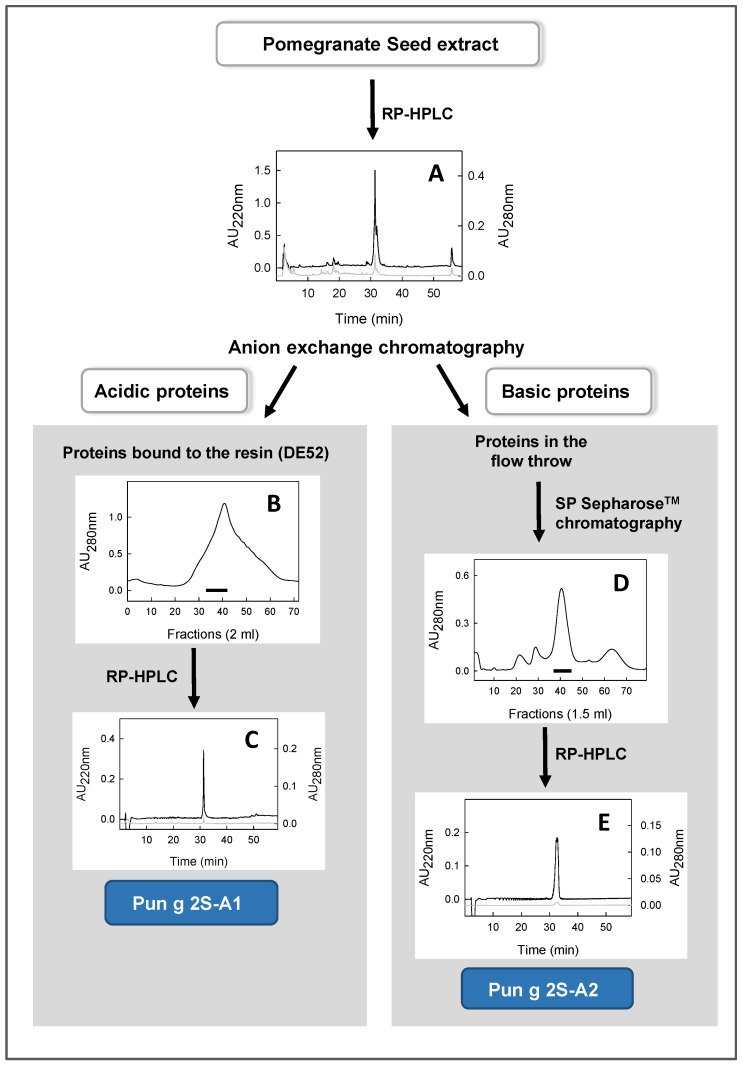
Schematic representation of the procedures used for the purification of the two 2S albumins from the pomegranate seed extract. (**A**) RP-HPLC profile of the pomegranate seed extract; (**B**) elution profile of the proteins previously bound to the DE52 resin; (**C**) RP-HPLC profile of purified Pun g 2S-A1; (**D**), elution from SP Sepharose resin of proteins previously collected in the flow throw of the DE52 resin; (**E**) RP-HPLC profile of purified Pun g 2S-A2. In subfigures (**A**,**C**,**E**), black line shows the absorbance at 220 nm, whereas grey line shows the absorbance at 280 nm. The black bars present in subfigures (**B**,**D**) indicate the pooled fractions containing the protein to be purified.

The seed extract was subjected to RP-HPLC chromatographic separation, shown in Figure 3A. The RP-HPLC profile shows a main peak eluted at about 32 min, which is not symmetrical and seems to contain at least two molecular species. Very small amounts of other components, causing small changes on the baseline, were also observed. The peak eluted at about 32 min was collected and analyzed on reducing SDS-PAGE, where major bands migrating at about 18 kDa and below 10 kDa were observed. On the basis of these results, the isolation of the proteins eluted at about 32 min via RP-HPLC was undertaken.

The extract was loaded on an anion exchange chromatographic column, made with the resin DE52, and two protein fractions were obtained, one eluted in the flow through and the other one bound to the resin (see the scheme in Figure 3). The proteins bound to this resin (acidic proteins) were eluted via a salt gradient. The chromatographic profile obtained by reading the obtained fractions at 280 nm is shown in Figure 3B. The fractions were analyzed via SDS-PAGE (and/or via RP-HPLC), and those showing a main band at about 18 kDa were pooled (see the black bar in Figure 3B) and further separated via RP-HPLC. This separation provided a fraction showing a single and narrow peak in the RP-HPLC chromatogram (Figure 3C). This protein was then identified as a 2S albumin, named Pun g 2S-A1.

The proteins eluted in the flow through (basic proteins) were further separated via cation exchange chromatography using the SP-Sepharose resin (Figure 3D). The eluted fractions were analyzed via RP-HPLC and/or SDS-PAGE, and those containing a peak at about 32 min were pooled (see the black bar in Figure 3D). Next, this pool was further separated via RP-HPLC. The peak eluted at about 32 min (Figure 3E) was manually collected and later identified as a 2S albumin, named Pun g 2S-A2.

### 3.3. SDS-PAGE Analysis of Purified Proteins

The electrophoretic behavior of purified acidic and basic proteins is shown in Figure 1. Under reducing conditions, the acidic protein showed a main band at an apparent molecular mass of about 18 kDa and a smear of bands at lower molecular masses (lane B), whereas in the absence of reducing agents, the same sample displayed a large band at about 16 kDa (lane D). The obtained data suggest that this protein is a monomer where some fragments of variable size are cut, but they are still held bound to the molecule, probably by disulfide bridges, in the absence of reducing agents. Under reducing conditions, instead, the disulfide bridges are opened and the cut fragments are released, in line with what is shown by the SDS-PAGE analysis (lane B).

The purified basic protein showed a large band in the absence of reducing agents at about 12 kDa (lane E). In contrast, in the presence of a reducing environment, it displayed a double band at an apparent molecular mass lower than 10 kDa (lane C), thus suggesting that this molecule is a heterodimer.

### 3.4. Identification of the Isolated Proteins as 2S Albumins

The purified acidic protein was subjected to N-terminal amino acid sequencing, which did not produce any result. This suggested that the N-terminal residue was blocked. Therefore, mass spectrometry was exploited for the identification. This methodology showed a single species with a molecular mass of 16,398.67 Da (Table 2).

**Figure 4:**
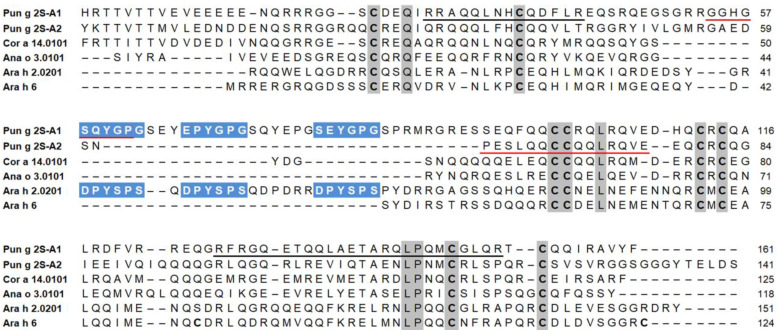
Multiple sequence alignment of 2S albumins without the signal peptide. The sequences of Pun g 2S-A1 and Pun g 2S-A2, Cor a 14.0101, Ana o 3.0101, Ara h 2.0201 and Ara h 6, having the UniProt accession numbers A0A218XU94, A0A2I0JHZ1, D0PWG2, Q8H2B8, Q6PSU2 and A5Z1R0, respectively, were aligned using the Clustal Omega algorithm, on the Expasy platform. A few manual displacements of the residues in the alignment were performed after a visual inspection. Residues conserved in all sequences are highlighted in gray, with cysteine residues in bold. The amino acid fragments obtained via mass spectrometry are underlined by black bars, whereas those derived via direct sequencing are indicated by red bars. Missing residues are shown by dashes. The three protein stretches (DPYSPS) reported as dominant epitopes in Ara h 2, and the corresponding fragments in Pun g 2S-A1, are highlighted in light blue with white characters.

In addition, it provided the amino acid sequence of two internal fragments, one of 14 residues (RRAQQLNHCQDFLR) and another one of 25 residues (RFRGQETQQLAETARQLPQMCGLQR) (Figure 4).

**Figure 5:**
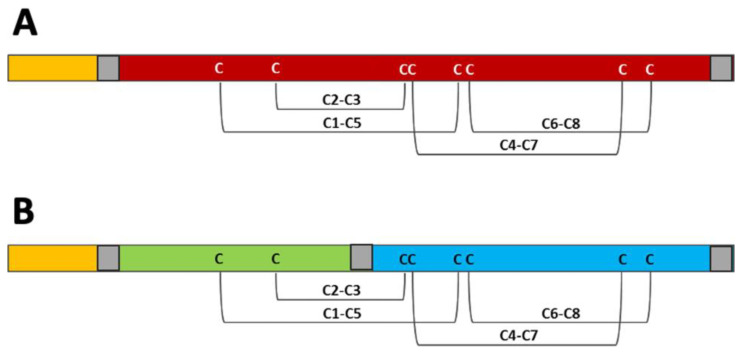
Schematic representation of Pun g 2S-A1 (**A**) and Pun g 2S-A2 (**B**), having the UniProt accession numbers A0A218XU94 and A0A2I0JHZ1, respectively. The disulfide bridge pattern formed between the eight conserved cysteine residues in the 2S albumin family is shown [31]. The signal peptide is shown in orange. In (**A**), the monomeric Pun g 2S-A1, without the signal peptide, is in bordeaux. In (**B**), the N-terminal small subunit and the C-terminal large subunit of the heterodimeric Pun g 2S-A2 are in green and light blue, respectively. The possible sites of proteolytic processing of the proteins (proforms) are shown in gray, but the scheme does not reflect the extension of the removed residues.

Moreover, this protein was digested using trypsin, the peptides were separated via RP-HPLC, and one of them was subjected to N-terminal sequencing. This experiment provided the amino acid sequence of an additional fragment (GGHGSQYGP). These three peptides were used to perform a similarity search in the UniProtKB, using the BLAST program. The bioinformatic analysis allowed the identification of this protein, which was named Pun g 2S-A1 (Figure 4), as the pomegranate seed 2S albumin having the UniProt accession number A0A218XU94. In fact, the three fragments revealed 100% of the sequence identity with internal regions of this protein. On the basis of the amino acid sequence, and after the signal peptide removal, the calculated molecular mass of the protein was 18,716.43 Da (Table 1). This value is 2317.76 Da higher than that (16,398.67 Da) obtained via the mass spectrometry experiments. In particular, in this case, the purified protein was shorter by about 19 residues, which were evidently removed from the protein present in the natural source. On the basis of the available literature [31], we could assume that some residues present in the protein proform, and removed in the protein form purified from the natural source, could be located partly at the N-terminal and partly at the C-terminal region of the molecule lacking the signal peptide (see the bordeux line in Figure 5A).

The purified basic protein was subjected to N-terminal amino acid sequencing which produced the sequence of a fragment of 15 residues: PESLQQCCQQLRQVE (Figure 4). The similarity search in the UniProt databank revealed 100% sequence identity with an internal fragment of one protein only, which is a 2S albumin from pomegranate seeds with accession number A0A2I0JHZ1. This protein was named Pun g 2S-A2. The analysis via mass spectrometry revealed a molecular mass of the entire molecule of 12,352.28 Da, which is 3814.69 Da lower than that calculated for the mature protein based on its amino acid sequence (16,116.97 Da). This result indicates that, in addition to the signal peptide, other amino acids had been removed. Next, the molecule was subjected to a reduction in the disulfide bridges followed by a further mass spectrometry analysis. The results revealed two molecules, one of 4013.69 Da (small subunit) and the other one of 8345.84 Da (large subunit). These results are in accordance with those obtained via SDS-PAGE and reveal that Pun g 2S-A2 is a heterodimer. On the basis of all the available data, it is conceivable to place the obtained PESLQQCCQQLRQVE sequence at the N-terminus of the large subunit (in light blue in Figure 5B). Then, if we consider that this subunit starts with the above sequence, we can calculate a theoretical molecular mass of 8890.97 Da, which is higher than the experimental one. However, if we cut the last five residues at the C-terminus (Figure 4), then the calculated molecular mass of the large subunit becomes 8345.42 Da, which is compatible with the experimental value (8345.84 Da) obtained via mass spectrometry. Therefore, we can conclude that the large subunit of Pun g 2S-A2, following the proteolytic processing, contains 74 amino acids, starting with PES and ending with GGY (Figure 4).

The small subunit of Pun g 2S-A2 corresponds to the region in green in Figure 5B. The assessment of the extension of the proteolytic processing occurring on the proform of the small subunit is more difficult because the N-terminus is not available via direct protein sequencing (blocked residue), and the C-terminus cannot be determined. Therefore, we do not know where the mature molecule starts and where it ends. Anyway, the experimental molecular mass of 4013.69 Da obtained via mass spectrometry, together with the assumption that the two cysteine residues contained in the small subunit are conserved, suggests that this subunit was subjected to proteolytic processing at both the N-terminus and the C-terminus.

### 3.5. Structural Investigation of Isolated Pomegranate 2S Albumins in Comparison with Allergenic Homologs from Other Sources

Figure 4 shows a multiple sequence alignment of 2S albumins made with the amino acid sequences found in the UniProt database using the accession numbers reported in Table 2. In particular, Pun g 2S-A1 and Pun g 2S-A2 are aligned with some homologs, registered as allergens by WHO/IUIS and included in the FABER^®^ test. They are the isoforms Cor a 14.0101 from hazelnut, Ana o 3.0101 from cashew, and Ara h 2.0201 and Ara h 6 from peanut. The isoforms to be aligned were selected as they resulted more similarly than others to the pomegranate 2S albumins, following the similarity search using the Allergome aligner tool on the Allergome platform.

The alignment shows that each 2S albumin has a peculiar size and a peculiar distribution of gaps, which are needed to align all the sequences. In particular, we can observe some large gaps in a central region, while others are a little smaller and placed at the N-terminus and C-terminus of some proteins. Figure 4 shows that, among the aligned sequences, the monomeric pomegranate Pun g 2S-A1 is the largest one. Compared to the other molecules, it lacks only a C-terminal region and shows a few gaps of one or two residues. It appears the most similar to the allergen, Ara h 2.0201, which seems to be a monomer [32] like pomegranate Pun g 2S-A1. In contrast, the heterodimeric Pun g 2S-A2 appears more similar to Cor a 14, Ana o 3 and Ara h 6, which have in common a large gap in the center, in addition to other lacking residues at the N-terminus and/or at the C-terminal region and other small scattered gaps.

These proteins are all rich in glutamine residues, but the amino acids conserved in the same position in all the sequences are very few. In fact, we can observe that they include the eight cysteines, and a few other residues (see the alignment in Figure 4). The two purified pomegranate proteins show a sequence identity of 46%, as obtained using the Clustal Omega algorithm on the Expasy platform. Both molecules have similarities with 2S albumins from different sources, such as Cor a 14.0101, Ana o 3.0101, Ara h 2.0201 and Ara h 6, with which Pun g 2S-A1 shares a sequence identity of about 45%, 31%, 28% and 26%, respectively, whereas Pun g 2S-A2 shows about 45%, 37%, 29% and 29% (Table 3).

### 3.6. Comparative Structural Investigations of Pomegranate 2S Albumins Found in UniProt Database

The two 2S albumins isolated from pomegranate seeds were compared with three additional molecules, from the same source, found in the UniProt database. Their alignment (Figure S1) shows a picture which is not very different compared to that of Figure 4, where the pomegranate 2S albumins are compared to homologs from other sources. In fact, it can be observed that each molecule has a peculiar size; some molecules lack the central region, whereas others lack the C-terminal and/or the N-terminal region. Table S1 shows the theoretical molecular mass and isoelectric point (pI), calculated by using the ProtParam tool on the basis of the protein sequence. It can be observed that the molecular mass of these (proform) 2S albumins ranges from 14,062.98 Da to 18,716.43 Da, and the pI ranges from 5.70 to 7.78. Figure S2 shows that the sequence identity between these five different pomegranate isoforms ranges from 30.65% to 54.89%.

### 3.7. IgE Binding Detection and Sensitization Profiles

Out of 703 Italian unselected patients reporting any type of allergy and subjected to the FABER^®^ test for the evaluation of specific IgEs in their sera, 28 (4.0%) were found to be sensitized to at least one of the six allergenic 2S albumins analyzed in this study (Table 4).

Pun g 2S-A1 and Pun g 2S-A2 were recognized by 1.7% and 0.28% of the tested patients, and only one subject had specific IgEs recognizing both the pomegranate proteins. Intermediate values, corresponding to 1.14%, 0.71%, 0.71% and 0.71%, were calculated for 2S albumins from other seeds, namely hazelnut (Cor a 14), cashew (Ana o 3), peanut (Ara h 2) and peanut (Ara h 6), respectively, that were simultaneously tested because they were available on the FABER^®^ biochip. Therefore, the highest frequency of sensitization was observed for pomegranate Pun g 2S-A1, whereas Pun g 2S-A2 from the same source showed the lowest IgE binding capacity.

## 4. Discussion

Preliminary experiments had shown that proteins recognized by specific IgEs are contained in the pomegranate seed extract [20]. As described in this paper, experiments of immunoblotting have confirmed the presence in pomegranate seeds of proteins recognized by IgE antibodies and showed the molecular mass of the reactive proteins, highlighting that the strongest signal could be associated with a protein band migrating at about 18 kDa. In fact, nine out of ten sera of patients allergic to pomegranate were revealed to contain specific IgEs able to bind the protein band at about 18 kDa. Two IgE-binding proteins, which appear as the main protein components of the seed, were isolated from the extract obtained from this pomegranate tissue. The protein profile of the extract suggests that the isolated molecules represent the main protein bands in the reducing SDS-PAGE and the main peaks in RP-HPLC separation. These protein components were analyzed via the combination of direct protein sequencing, bioinformatics and mass spectrometry experiments, and identified as belonging to the 2S albumin family, and were named Pun g 2S-A1 and Pun g 2S-A2.

Several studies have reported that 2S albumins are responsible for severe allergic reactions to nuts, seeds and legumes, making these proteins clinically relevant for allergic sensitization and as potential diagnostic markers [33,34]. In order to investigate the possibility that pomegranate 2S albumins are potentially responsible, or at least partially, for the immunological results reported in the literature for seeds [16], and observed in this study via immunoblotting, the purified molecules were tested for IgE binding following immobilization on the FABER^®^ biochip, as experimental molecules. The obtained results confirmed that both the pomegranate seed 2S albumins could be recognized and bound by specific IgEs contained in the sera of suspected allergic patients, although the two proteins showed peculiar behaviors. In line with this observation, a different frequency of IgE binding with the two pomegranate 2S albumins was observed in the analyzed Italian population of 703 suspected allergic patients. In agreement with the immunoblotting results, Pun g 2S-A1, showing a band at about 18 kDa, following the FABER^®^ test is recognized by a specific IgE with a higher frequency, and with stronger signals, than Pun g 2S-A2, which migrates as a double band at a molecular mass lower than 10 kDa in reducing SDS-PAGE. In particular, Pun g 2S-A1 and Pun g 2S-A2 were recognized in 1.7% and 0.28%, respectively, of the tested 703 patients, and only one subject had specific IgEs for both the pomegranate proteins. Some intermediate frequencies were calculated for 2S albumins from other seeds, such as that from hazelnut (Cor a 14), cashew (Ana o 3), peanut (Ara h 2) and (Ara h 6), that were available on the FABER^®^ biochip and simultaneously tested.

Many 2S albumins contained in several plant-based foods have been described as major allergens for some populations [35], whereas the frequency of sensitization to 2S albumins appears low in the Italian population analyzed via the FABER^®^ test. This observation can be placed in the context of the literature reports highlighting the variations of sensitization to some allergens associated with different populations [36]. For instance, it is known that sensitization and allergy to LTP have a higher prevalence in the Mediterranean countries, such as Italy, than in other places [37,38], such as northern Europe, where other allergens, like pathogenesis-related protein PR-10 and seed storage proteins, including 2S albumins, are reported to have a higher frequency [39].

The different immunological behaviors of Pun g 2S-A1 and Pun g 2S-A2 are associated with the different structural features of these two proteins, although their very close chromatographic elution from RP-HPLC might suggest that they are similar molecules. They were purified following two different procedures, because they had a different chromatographic behavior on ionic resins. In fact, in line with their different theoretical pI values, that were acidic for Pun g 2S-A1 and a little basic for Pun g 2S-A2, these proteins behaved like an anion and a cation, respectively, in the applied experimental conditions. This observation suggests that, depending on the purification procedure, different 2S albumin isoforms can be purified from an allergenic source, as observed here for pomegranate. Actually, in each species, 2S albumins represent a multigenic family [40,41,42,43] coding for a group of isoforms characterized by a few common features and several structural and physicochemical differences. In fact, similar to other 2S albumins [44,45,46,47], the pomegranate homologs have a blocked N-terminal residue and are rich in arginine and glutamine, representing a great source of nitrogen, and methionine, which is essential for sulfur storage.

Beyond that, the two 2S albumins purified from pomegranate seeds show important structural differences, which are in line with the literature about this protein family. It is reported that 2S albumins are synthesized as a single polypeptide with a theoretical molecular mass ranging from 18 to 24 kDa, that undergoes several post-translational modifications, including proteolysis, signal peptide removal and the formation of disulfide bridges [48,49]. In most 2S albumins, the precursor, or protein proform, is further cleaved into two subunits by an aspartic protease, thus forming a heterodimer which comprises a small subunit of about 3–4 kDa and a large one of about 8–10 kDa, kept together by two disulfide bonds [43,47,50]. A few monomeric 2S albumins have also been reported [41,51]. Further post-translational modifications can be found, such as glycosylation, N-terminal cyclization of glutamine residues, phosphorylation of serine and threonine residues, and cleavages at the N- and C-terminal ends of the monomer or of both subunits [31,52,53]. Many of these modifications have been detected in one or both the isolated pomegranate 2S albumins. First of all, it can be observed that Pun g 2S-A1 is monomeric, whereas Pun g 2S-A2 is a heterodimer. In agreement with this observation, the mass spectrometry results demonstrated that the molecular mass of mature Pun g 2S-A1 is compatible with a monomer of about 16 kDa. This value is lower than that calculated on the basis of the amino acid sequence found in the UniProt databank and of the migration in SDS-PAGE (about 18 kDa). Moreover, the smears observed at a lower molecular mass in reducing SDS-PAGE, and around the main band in the absence of reducing agents, might be interpreted by assuming the occurrence of molecular heterogeneity, already reported in the literature [31,41,54]. In fact, this can be due to non-homogeneous proteolytic processing of originating fragments that can be released when the disulfide bridges are opened in the reducing environment. In contrast to Pun g 2S-A1, on the basis of SDS-PAGE, Pun g 2S-A2 results in being a heterodimer showing a doublet at a low molecular mass in a reducing environment and a single large band, migrating at a higher value of molecular mass, in the absence of reducing agents. The mass spectrometry experiments confirmed that this molecule is a heterodimer and that the entire molecule has a molecular mass of 12,352.28 Da, but the reduction of the disulfide bridges causes the splitting into two molecules of 4013.69 and 8345.84 Da, in agreement with the molecular mass of the doublet observed in reducing SDS-PAGE. In addition, it can be observed that the molecular mass of this molecule is lower than that calculated on the basis of the amino acid sequence found in UniProt, thus indicating that processing events, in addition to the removal of the signal peptide, occurred.

A low sequence identity characterizes these two purified pomegranate 2S albumins isoforms when they are compared to each other, as observed also for the additional three homologs from the same source, found in the database. The highest identity value shown by these proteins was 54.89%, whereas higher values (generally above 80%, and in any case above 69%) were reported when the isoforms of plant allergenic proteins belonging to other families, such as Bet v 1 [28], or the papain-like cysteine protease Ana c 2 [55], were compared. Actually, a low identity between protein sequences was observed also when Pun g 2S-A1 and Pun g 2S-A2 were compared with the homologous proteins from the seeds of other plant sources, such as hazelnut, cashew and peanut. In line with this observation, a multiple sequence alignment of 2S albumins from these sources reveals that only a few residues are conserved in all these proteins, and most of them (eight residues) are represented by cysteines, known to be involved in disulfide bridges which stabilize the three-dimensional structure [56,57].

The literature reports that, despite the low amino acid sequence identity, all 2S albumins have a conserved three-dimensional structure comprising five α-helices (two in the region of the small subunit and three in the region of the large one) arranged in a right-handed superhelix. In the case of the heterodimeric structure, it is fastened through two inter-chain and two within-the-large-subunit disulfide bridges [56,57].

Monomeric 2S albumins, like Ara h 2, have a central region (linker) connecting the N-terminal and C-terminal regions, which is missing in heterodimeric proteins. The pomegranate monomeric 2S albumin, Pun g 2S-A1, has a linker of about 30 residues that is absent in Pun g 2S-A2 and in other homologs, except in allergens such as Ara h 2.0201. However, the sequence of this region of Ara h 2.0201 appears poorly conserved when compared with that of Pun g 2S-A1. In this region, the peanut allergen displays three copies of the motif DPYSPS, which is described as an immunodominant IgE epitope in Ara h 2 [58,59]. Intriguingly, the three amino acid sequence stretches can be aligned with a quite similar motif found in the Pun g 2S-A1 linker. In particular, the last four residues of the Ara h 2 motif, that is YSPS, can be aligned with the Pun g 2S-A1 YGPG, where Y and P are conserved in the same position and the S, repeated in Ara h 2, is substituted by G, repeated in the pomegranate homolog. The two residues preceding YSPS in Ara h 2 are DP, whereas those preceding YGPG in Pun g 2S-A1 are EP, SE or SQ. The high immunological importance of the motif found in Ara h 2 has already been demonstrated [59,60], whereas the relevance of the corresponding sequences in the pomegranate monomeric 2S albumin is not clear, and needs to be further investigated. Probably, additional studies could be performed by analyzing populations with different features, including eating habits and exposure to aeroallergens [36,39]. Therefore, the description of the relevance of this pomegranate 2S albumin as a cause of allergic reactions should be postponed until other data appropriate for the purpose become available.

Indeed, these structural motifs are absent in Pun g 2S-A2. However, it is well known that several IgE epitopes are present also in heterodimeric 2S albumins, which lack the so-called linker region, but are still also capable of causing severe allergic symptoms [61,62]. This is the case of 2S albumins such as Cor a 14, Ana o 3 and Ara h 6, which are considered relevant allergens [32,63,64,65] and cause sensitization with a higher frequency than the pomegranate Pun g 2S-A2, also in the analyzed Italian population. Therefore, also in the case of Pun g 2S-A2, its real relevance as a cause of allergic reactions should be delayed until additional data become available.

In conclusion, this study demonstrated that the 2S albumins available in pomegranate seeds are recognized by specific IgEs. The protocols to isolate two 2S albumins were set up and now can be used to purify them from a pomegranate seed extract and extend their characterization. In particular, the two isolated proteins consist of a monomeric one, showing some structural similarities with the well-known peanut allergen, Ara h 2, and a heterodimeric one, like Cor a 14, Ara h 6 and Ana o 3. The real relevance in clinical allergology is yet to be established. For this purpose, it will be very important to extend the investigation to populations with different features. Nevertheless, this study can be considered as a starting point because it provides initial data and protocols to obtain the pure proteins to be used for future more extensive and in-depth investigations.

## Figures and Tables

**Table 1:** History of pomegranate allergens.

Date	Mr (kDa)	Tissue	Allergen	Bibliography
1999	29	pulp	Not identified	[17]
2007	9	pulp	Pun g 1	[18]
2017	7	pulp	Pun g 7	[19]
2018	29	Pulp and seeds	Pun g 14	[20]

**Table 2:** Details of 2S albumins aligned in Figure 4. The theoretical information was referenced from the amino acid sequences found in UniProt, without the signal peptide, and using the indicated accession numbers.

Protein/Allergen Name	Protein Accession Number (in UniProt)	Theoretical ^a^ Mr (Da)	Experimental Mr (Da) ^b^	Theoretical pI ^a^
Pun g 2S-A1	A0A218XU94	18,716.43	16,398.67	7.74
Pun g 2S-A2	A0A2I0JHZ1	16,116.97	12,350.50	6.77
Cor a 14.0101	D0PWG2	14,916.49	N/A	5.61
Ana o 3.0101	Q8H2B8	14,234.74	N/A	5.37
Ara h 2.0201	Q6PSU2	17,993.59	N/A	5.51
Ara h 6	A5Z1R0	14,845.61	N/A	5.49

^a^ Mr and pI were calculated using the software ProtParam, on the Expasy platform. ^b^ Experimental data were from mass spectrometry. N/A stands for “not available”.

**Table 3:** Amino acid sequence identity (%) between the 2S albumins shown in the alignment of Figure 4. The identity values were calculated using the Clustal Omega algorithm on the UniProt website, by comparing the amino acid sequences found in UniProt, without the signal peptide (proform protein), two by two.

	Pun g 2S-A1	Pun g 2S-A2	Cor a 14.0101	Ana o 3.0101	Ara h 2.0201	Ara h 6
Pun g 2S-A1	100					
Pun g 2S-A2	46.03	100				
Cor a 14.0101	45.16	44.80	100			
Ana o 3.0101	31.30	36.84	41.23	100		
Ara h 2.0201	28.17	28.81	28.83	21.93	100	
Ara h 6	26.05	28.70	33.33	22.52	60.66	100

**Table 4:** Sensitization to 2S albumins of 28 out of 703 Italian patients analyzed using the FABER^®^ test.

Patients		FABER^®^ 244 (FIU) ^a^					
N°	Gender	Pun g 2S-A1	Pun g 2S-A2	Cor a 14	Ana o 3	Ara h 2	Ara h 6
1	F	0.5	0	0	0	0	0
2	F	1.0	0	0	0	0	0
3	F	0.9	0	0	0	0	0
4	F	0.7	0	0	0	0	0
5	F	0.5	0	0	0	0	0
6	F	4.3	0	0	0	0	0
7	M	0.2	0	0	0	0	0
8	M	0.5	0	0	0	0	0
9	M	1.3	0	0	0	0	0
10	M	0.5	0	0	0	0	0
11	M	14.3	0	0	0	0	14.3
12	F	2.6	2.4	3.8	1.8	2.0	1.3
13	F	0	0.7	0	0	0	0
14	F	0	0	1.2	0	0	0
15	M	0	0	0	1.4	0	0
16	F	0	0	0	3.8	0	0
17	M	0	0	0	2.6	0	0
18	M	0	0	67.0	27.8	91.0	22.9
19	M	0	0	0	0	4.3	0
20	F	0	0	0	0	1.6	0
21	F	0	0	0	0	0.4	0
22	F	0	0	0	0	0	1.0
23	F	0	0	0	0	0	0.4
24	F	0	0	1.2	0	0	0
25	F	0	0	8.9	0	0	0
26	F	0	0	0.2	0	0	0
27	M	0	0	5.5	0	0	0
28	M	0	0	3.9	0	0	0

^a^ FIU, FABER^®^ International Units, positive value FIU ≥ 0.01.

## Data Availability

The original contributions presented in the study are included in the article/Supplementary Material; further inquiries can be directed to the corresponding author.

## Supplementary Materials

The following supporting information can be downloaded at: https://www.mdpi.com/article/10.3390/foods13131965/s1, Table S1. Details of pomegranate 2S albumin isoforms found in the UniProt database; Figure S1. Multiple sequence alignment of pomegranate 2S albumin isoforms as obtained via Clustal Omega, on the Expasy platform; Figure S2. Identity (%) between the five pomegranate 2S albumin sequence isoforms obtained via the Clustal Omega software on the Expasy platform.
