# Supplementary material for: Isolation, Characterization and IgE Binding of Two 2S Albumins of Pomegranate Seeds
Source: Foods. 2024 Jun 21;13(13):1965. doi: 10.3390/foods13131965 (PMC11241328; doi:10.3390/foods13131965)
Supplement: Supplementary file 1 [file foods-13-01965-s001.zip › foods-3061427-supplementary.pdf]

ONLINE REPOSITORY

Isolation, characterization and IgE binding of two 2S albumins of pomegranate seeds

Lisa Tuppo [1], Claudia Alessandri [2], Laura Zaccaro [3], Ivana Giangrieco [1], Maurizio Tamburrini [1], Adriano Mari [2], Maria Antonietta Ciardiello [1,*]

[1] Institute of Biosciences and BioResources (IBBR), National Research Council of Italy (CNR), 80131 Naples, Italy; lisa.tuppo@ibbr.cnr.it (L.T.); ivana.giangrieco@ibbr.cnr.it (I.G.); maurizio.tamburrini@ibbr.cnr.it (M.T.)

[2] Associated Centers for Molecular Allergology (CAAM), 00100 Rome, Italy; claudia.alessandri@caam-allergy.com (C.A.); adriano.mari@caam-allergy.com (A.M.)

[3] Institute of Biostructures and Bioimaging, National Research Council of Italy (CNR), 80131, Naples, Italy; laura.zaccaro@cnr.it (L.Z.)

* Correspondence: mariaantonietta.ciardiello@ibbr.cnr.it;

Table S1. Details of pomegranate 2S albumin isoforms found in UniProt database

Isoform accession number	Theoretical Mr (Da)	Theoretical pI
A0A218XU94	18716.43	7.74
A0A2I0JHZ1	16116.97	6.77
A0A218XVS0	15803.58	5.70
A0A218XUM2	15717.75	6.96
A0A218XUV4	14062.98	7.78

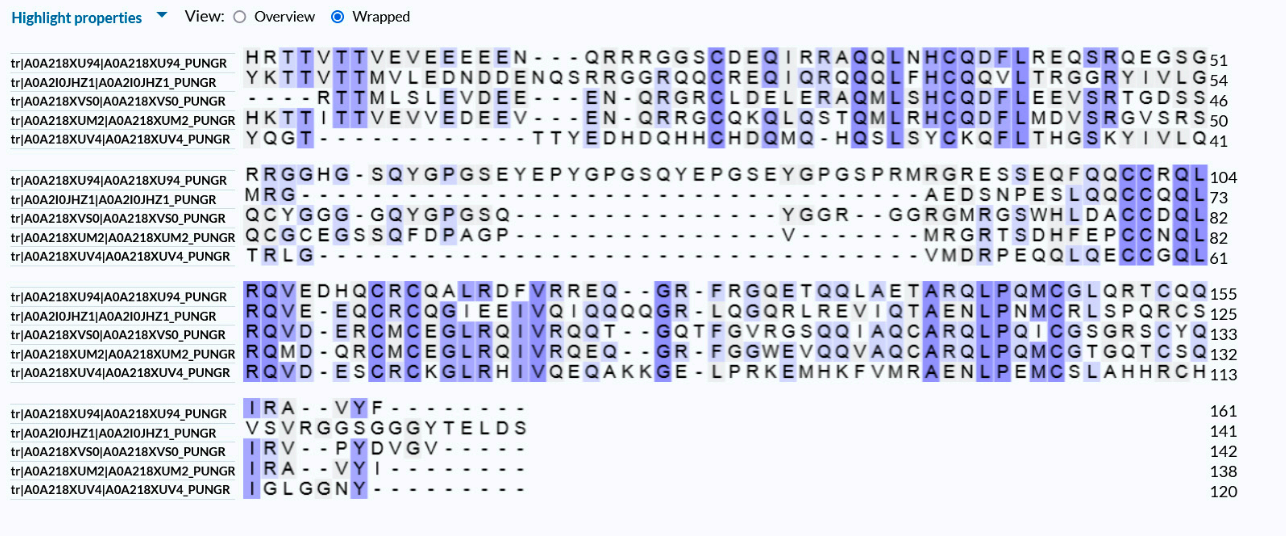

Figure S1. Multiple sequence alignment of pomegranate 2S albumin isoforms as was obtained by Clustal O, on the Expasy platform. The residues conserved in all the sequences are highlighted in dark blu, those conserved in most of the sequences are highlighted in light blue. Missing residues are shown with dashes.

Percent Identity Matrix

tr|A0A218XU94|A0A218XU94_PUNGR	100.00%	41.27%	47.10%	50.36%	31.86%
tr|A0A2I0JHZ1|A0A2I0JHZ1_PUNGR	41.27%	100.00%	30.65%	31.20%	41.18%
tr|A0A218XVS0|A0A218XVS0_PUNGR	47.10%	30.65%	100.00%	54.89%	34.26%
tr|A0A218XUM2|A0A218XUM2_PUNGR	50.36%	31.20%	54.89%	100.00%	31.25%
tr|A0A218XUV4|A0A218XUV4_PUNGR	31.86%	41.18%	34.26%	31.25%	100.00%

Figure S2. Identity (%) between the five pomegranate 2S albumin sequence isoforms obtained by Clustal Omega software on the Expasy platform.
